# Pen Fouling in Finisher Pigs: Changes in the Lying Pattern and Pen Temperature Prior to Fouling

**DOI:** 10.3389/fvets.2019.00118

**Published:** 2019-04-16

**Authors:** Mona Lilian Vestbjerg Larsen, Maja Bertelsen, Lene Juul Pedersen

**Affiliations:** Department of Animal Science, Aarhus University, Aarhus, Denmark

**Keywords:** pen fouling, finisher pigs, early detection, lying behaviour, pen temperature

## Abstract

Pen fouling, where the pigs choose to rest in their designated excretion area (the slatted floors) and excrete in their designated resting area (the solid floors), is an undesired behaviour and should be prevented when possible. One strategy to prevent fouling is early detection by means of either animal or environmental measures changing prior to fouling. The aim of the present study was to investigate whether the lying pattern of pigs and the temperature in the pen changed the last 5 days prior to an event of fouling and whether this differed from pens without an event of fouling (controls). Fouling events was recorded at pen level when at least half of the solid floor was wet with excreta and/or urine (day0). Each fouling pen was paired with a control pen that had not been scored as a fouling pen prior to or at least 1 week after the fouling event. Fouling and control pens were either not provided with straw or provided daily with 150 g of straw per pig. Percentage of pigs lying on the solid floor and the slatted floor (36 events) as well as pen temperature above the solid and slatted floor (24 events) was analysed using four linear mixed effects models. The percentage of pigs lying on the solid floor decreased (40–24%; *P* < 0.05) while the number of pigs lying on the slatted floor increased (14–24%; *P* < 0.05) from day-2 to day0 only in the fouling pens, with differences seen between fouling and control pens on the same days (*P* < 0.01). However, these changes and differences was only seen in pens without straw. Also only in pens without straw did pen temperature above the solid floor decrease from day-2 to day0 (18.6–17.6°C; *P* < 0.001), with differences seen between fouling and control pens only on day0 (*P* < 0.05). In contrast, pen temperature measured above the slatted floor did not change, independent of whether the pen was provided with straw or not. Thus, in pens not provided with straw, both the lying pattern of pigs and pen temperature above the solid floor have potential as early detectors of pen fouling.

## Introduction

Housing pigs in pens with partly slatted floors is considered both a welfare and environmental improvement compared to pens with fully slatted floors. Pigs prefer to lie on a solid surface (1). Also, the manure surface, and thereby the ammonia emission, will be decreased in pens with partly solid floors. However, pigs in pens including a solid surface are at risk of developing pen fouling. Pen fouling happens when the pigs change their lying and excretory behaviour away from their designated areas, i.e., when the pigs start lying on the slatted surface and excrete on the solid surface. Pen fouling results in lower hygiene, worse air quality, higher ammonia emissions, higher workload for the farmer, disturbance of the pigs' resting behaviour and an increase in agonistic interactions between the pigs (1–3). Thus, the prevention of pen fouling is important. However, pen fouling is a multifactorial problem, and risk reduction alone can be an efficient but difficult approach in order to prevent fouling. However, if combined with early warning of fouling at pen level, the farmer will be able to do pen specific early interventions based on the risk factors present in the pen. Often, pen fouling occurs due to an insufficient thermal climate, and prevention strategies include the activation of sprinklers and an increase in the airflow. Therefore, the early detection strategy would also make it possible for the farmer to change the temperature curve dynamically in time to accommodate the needs of the pigs. One of the major factors identified to affect fouling is the thermal climate of the pen, including temperature, humidity, draught and overall heat balance of the pigs (4). Thus, changes in pen temperature or other climate parameters may work as early detectors of pen fouling. As excretion in the solid area is usually accompanied with a change in lying area, changes of pigs' lying pattern may also work as an early detector of pen fouling. However, to work as early detectors, changes in pen temperature and lying pattern need to occur prior to the registration of a fouling event.

The aim of the present study was to investigate whether changes in pigs' lying pattern and pen temperature occur prior to fouling and whether these differ from what is seen in pens without fouling. It was hypothesised that the pigs would lie less on the solid floor and more on the slatted floor prior to fouling and that pen temperature measured in the solid area of the pen would increase prior to fouling.

## Materials and Methods

The study was conducted from June 2015 to November 2016 in accordance with a protocol approved by the Danish Animal Experiments Inspectorate (Journal no. 2015-15-0201-00593) and included in total four batches of finisher pigs from 30 kg until slaughter, previously described in Larsen et al. (5). The current study includes a subset of those pens scored as fouling pens (*n* = 36) and their respective control pens (*n* = 36). A pen was scored as a fouling pen if more than half of the solid floor was wet with excreta and/or urine (day0), while a control pen had not been scored as a fouling pen prior to or at least 1 week after the fouling event in the fouling pen. Fouling and control pens had the same combination of each level of two treatments: (1) **STRAW**: not provided with straw (*n* = 34) or provided daily with 150 g of straw per pig on the solid floor (*n* = 38), (2) **STOCK**: initial space allowance of 0.73 m^2^/pig (18 pigs, *n* = 44) or 1.21 m^2^/pig (11 pigs, *n* = 28). Whether a pen could be scored as a fouling pen was recorded daily between 10:00 and 12:00 h by trained stock people. Only the first fouling event for each fouling pen was included.

### Housing and Management

The study was conducted in the experimental pig facilities at the Department of Animal Science, Aarhus University, Denmark, and was part of a larger study on risk factors for and early detection of tail biting (5). The facilities included two finisher pig units including 16 pens each with identical dimensions of 2.48 × 5.45 m (13.52 m^2^). The floor of the pens was divided between one third of solid, drained and slatted flooring. The gap between the slats was 2 cm for both the drained and slatted floor, whereas the slats were 8 cm wide for the slatted floor and 18 cm wide for the drained floor. The temperature curve used by the automated ventilation system to adjust the climate according to the weight of the pigs (SKOV A/S, Roslev, DK) decreased from 21°C in week 1 after insertion to 17°C in week 8 and onwards. The climate in the sections was, as a supplement to the automated control, evaluated each morning and adjusted according to the needs of the pigs (if the pigs was seen lying in the slatted floor instead of the solid floor from video before entering the section) by a parallel shift of the temperature curve of 0.5–1.5°C up or down. Each pen included an automatically controlled sprinkler system (SKOV A/S, Roslev, DK) above the slatted floor. This was turned on the whole time during all rounds from 08:00 to 20:00 h, except if the outdoor temperature fell below 5°C. The sprinkler system followed a linear curve going from 1% at a 0.5–°C increase from the temperature curve to 100% at a 4.0–°C increase. At 1%, the sprinklers were turned on with 45 min' intervals for 1 min and at 100% with 20 min' intervals for 3 min. In the current study, the minimum was 14%. The pigs were fed *ad libitum* with a commercial dry feed (15.1–15.5% crude protein), and the feeder was filled each day at 03:00, 10:00, and 18:30 h. Each pen included one dry feeder with either three or two feeding spaces, depending on the initial group size, separated by solid sides. Artificial light was on from 05:30 to 18:30 h. The pigs were raised according to standard Danish practices and by trained stock people.

### Behavioural Observations and Sensor Data

One camera (Monacor, TYPE-TVCCD-170S, Bremen, Germany) was installed approx. 3 m above the solid floor in each of the pens. The camera was placed close to the back wall of the pen, providing a full view of the entire pen. Three observers (two for batch 1–3, one for batch 4) scored the videos for the number of pigs lying on each type of floor (solid, drained and slatted) by instantaneous sampling every 10 min from 06:00 to 08:00 h and from 12:00 to 14:00 h on the last 5 days prior to a fouling event and on the day of the fouling event in each fouling pen. These two observation periods each day was chosen as these were quiet periods with no disturbances from feeding or people in the finisher sections. The control pens were observed on the same days and times as their respective fouling pen. Prior to analysis, data were aggregated to the average percentage of pigs lying in the pen, lying on the solid floor and lying on the slatted floor within each observation period on each day.

Pen temperature was measured by temperature sensors placed in two locations of the pen: one 63 cm above the solid floor and one 53 cm above the slatted floor, both placed on pen walls. Pen temperature was recorded every second. However, prior to analysis, it was aggregated first to average pen temperature for each hour of the day and next to average pen temperature for each day on both the solid and the slatted floor.

### Statistical Analysis

All statistical analyses were performed in R Version 3.4.3. (6), and all models were reduced using stepwise backward selection according to a 5% significance level (*P* < 0.05).

The responses investigated were the percentage of pigs lying in the pen, on the solid floor and on the slatted floor, all three including 36 fouling events. In addition, average daily pen temperatures above the solid and slatted floor were investigated, both including 24 fouling events. The difference in number of events between the responses was caused by missing data due to failure in either camera or temperature sensors. Thus, the events included in the data on pen temperature are not always the same as the events included in the data on pigs' lying pattern. Prior to analysis, the percentage of pigs lying in the pen was square transformed, and the percentage of pigs lying on the slatted floor was square root transformed to ensure compliance with the assumptions of the Gaussian (normal) distribution.

All responses were analysed with Gaussian linear mixed effect models, including the main effects: Group (control v. fouling pen), Obsday (−5 to 0) as categorical, Period (morning: 06:00–08:00 h v. afternoon: 12:00–14:00 h; only for lying pattern), STRAW (no v. yes), TotalPigs (group size on the actual day) and Age (age of the pigs at day0, ranged from day 14–69). The models also included all two and three-way interactions between Group, Obsday and STRAW. Further, the models allowed for a separate intercept for each Obsday nested within Pen number, Event number and Batch number (1–4).

## Results

### Lying Pattern of the Pigs

A generally higher percentage of pigs was seen lying in the morning compared to the afternoon period [84 vs. 76%; *F*_(1, 426)_ = 423.05; *P* < 0.001] and increased with an increase in age of the pigs [*F*_(1, 31)_ = 45.30; *P* < 0.001]. However, the percentage of pigs lying did not change prior to an event of fouling and did not differ between fouling and control pens.

**Figure 1:**
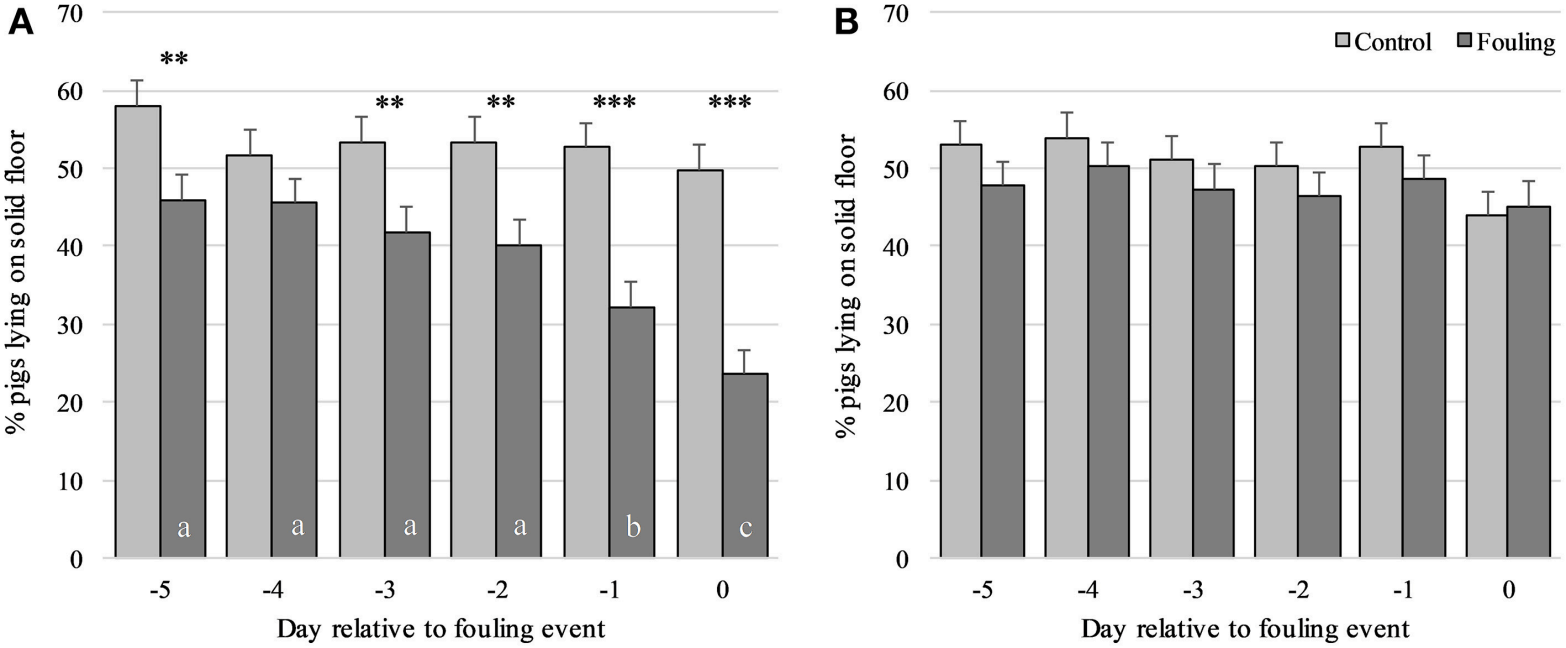
Percentage of pigs lying on the solid floor from day-5 to day0 relative to an event of fouling in pens with no straw provided **(A)** and in pens provided daily with 150 g of straw per pig on the solid floor **(B)** divided between control and fouling pens. **P < 0.01 and ***P < 0.001 indicate differences between control and fouling pens. Difference in lower case letters (a, b, c) indicates differences between observation days within each pen type (control and fouling).

For the percentage of pigs lying on the solid floor, a three-way interaction was found between Group, Obsday and STRAW [*F*_(10, 340)_ = 5.13; *P* < 0.001]. This interaction revealed a lower percentage of pigs lying on the solid floor in the fouling pens compared to the control pens on all observation days (except day-4), but only for pens without straw (Figure 1A). No differences were found between fouling and control pens when provided with straw (Figure 1B). The percentage of pigs lying on the solid floor also decreased prior to day0, but again only for pens without straw (Figure 1A). In the control pens, the percentage of pigs lying on the solid floor did not change prior to day0, irrespective of whether they were provided with straw or not. For fouling pens only, a higher percentage of pigs lying on the solid floor was seen in pens with straw compared to pens without straw on day-1 (*P* < 0.001) and day0 (*P* < 0.001). A higher percentage of pigs lying on the solid floor was seen in the morning compared to the afternoon period [*F*_(1, 424)_ = 176.99; *P* < 0.001] and decreased with an increase in group size [*F*_(1, 424)_ = 15.84; *P* < 0.001] and age of the pigs [*F*_(1, 30)_ = 6.96; *P* < 0.05].

**Figure 2:**
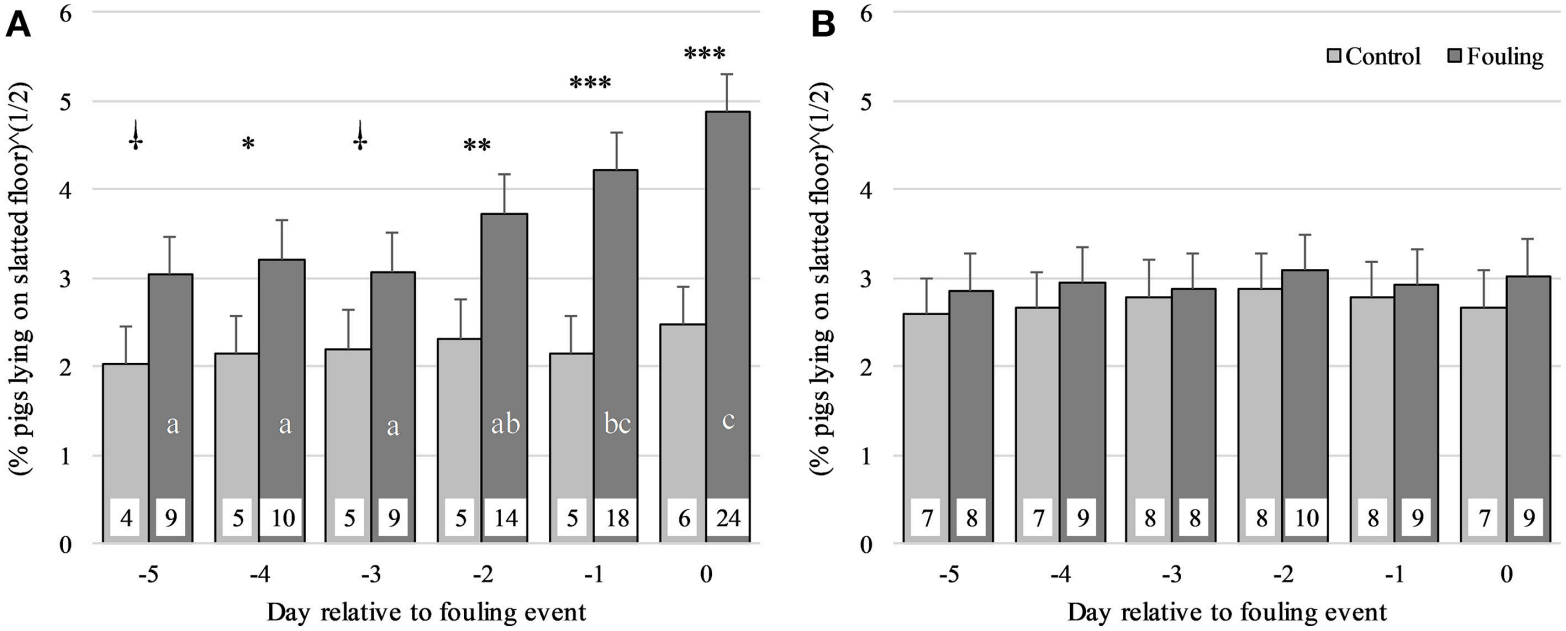
Percentage of pigs lying on the slatted floor from day-5 to day0 relative to an event of fouling in pens with no straw provided **(A)** and in pens provided daily with 150 g of straw per pig on the solid floor **(B)** divided between control and fouling pens. ^
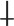
^*P* < 0.1, *P < 0.05, **P < 0.01 and ***P < 0.001 indicate differences between control and fouling pens. Difference in lower case letters (a, b, c) indicates differences between observation days within each pen type (control and fouling).

For the percentage of pigs lying on the slatted floor, a three-way interaction was found between Group, Obsday and STRAW [*F*_(5, 340)_ = 2.61; *P* < 0.05]. This interaction revealed a higher percentage of pigs lying on the slatted floor in the fouling pens compared to the control pens on all observation days (only tendencies on day-5 and day-3), but only for pens without straw (Figure 2A). No differences were found between fouling and control pens when provided with straw (Figure 2B). The percentage of pigs lying on the slatted floor also increased up to day0, but again only for pens without straw (Figure 2A). In the control pens, the percentage of pigs lying on the slatted floor did not change up to day0, irrespective of whether they were provided with straw or not. For the fouling pens only, a lower percentage of pigs lying on the slatted floor was seen in pens with straw compared to pens without straw on day-1 (*P* < 0.05) and day0 (*P* < 0.01). A generally lower percentage of pigs lying on the slatted floor was seen in the morning compared to the afternoon period [*F*_(1, 426)_ = 45.97; *P* < 0.001] and increased with the age of the pigs [*F*_(1, 30)_ = 12.00; *P* < 0.01].

### Pen Temperature

**Figure 3:**
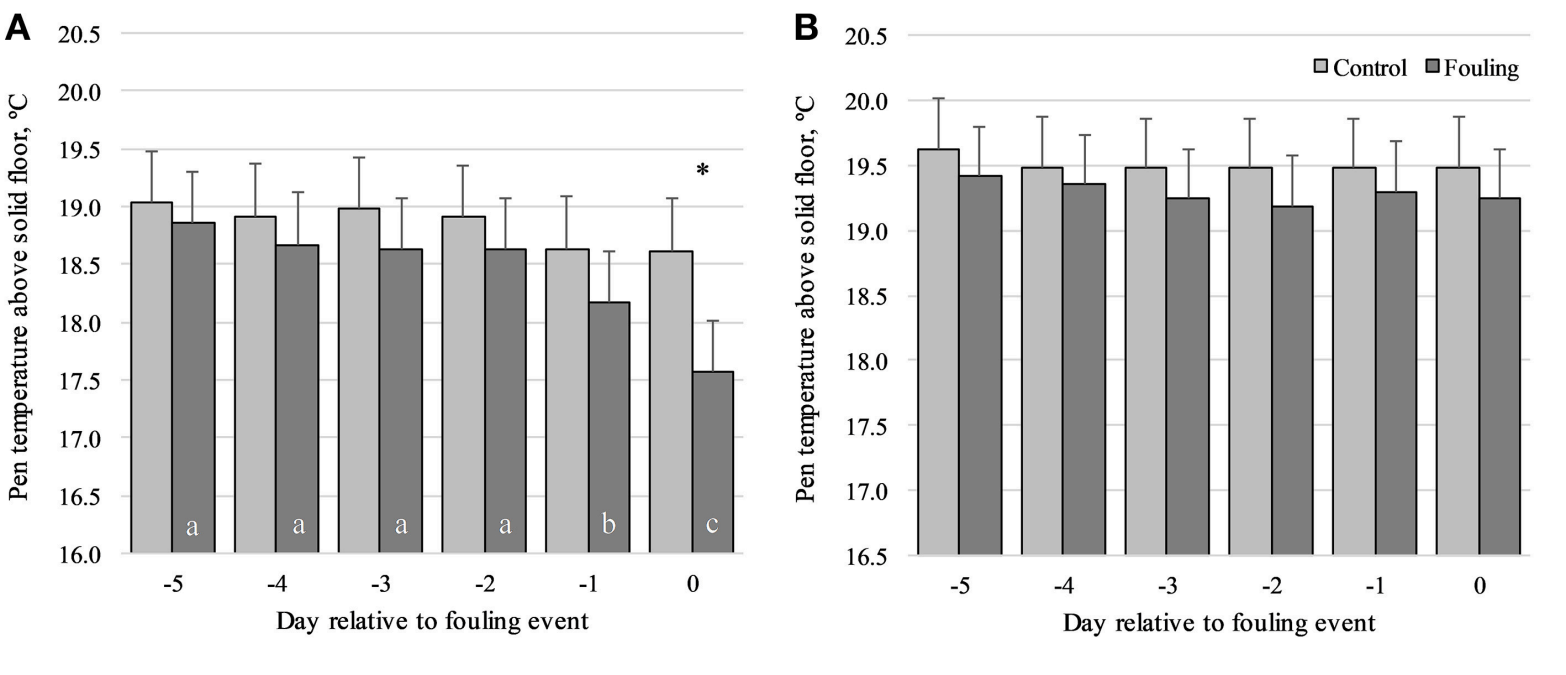
Pen temperature above the solid floor from day-5 to day0 relative to an event of fouling in pens with no straw provided **(A)** and in pens provided daily with 150 g of straw per pig on the solid floor **(B)** divided between control and fouling pens. *P < 0.05 indicates differences between control and fouling pens. Difference in lower case letters (a, b, c) indicates differences between observation days within each pen type (control and fouling).

For pen temperature above the solid floor, a three-way interaction was found between Group, Obsday and STRAW [*F*_(5, 220)_ = 2.26; *P* < 0.05]. This interaction revealed a lower pen temperature above the solid floor in fouling pens compared to control pens on day0 (1.05°C; *P* < 0.05), but only for pens without straw (Figure 3A). No differences were found between fouling and control pens when also provided with straw (Figure 3B). Pen temperature above the solid floor also decreased prior to day0, but again only for pens without straw (Figure 3A). In the control pens, pen temperature above the solid floor did not change prior to day0, irrespective of whether they were provided with straw or not. For fouling pens only, a lower pen temperature above the solid floor was found in pens without straw compared to pens with straw on day-1 (1.13°C; *P* < 0.05) and day0 (1.68°C; *P* < 0.01). Pen temperature above the solid floor increased with an increase in group size [*F*_(1, 219)_ = 22.75; *P* < 0.001] and decreased with an increase in age [*F*_(1, 20)_ = 13.34; *P* < 0.01].

Pen temperature above the slatted floor was on average 17°C (range 16.8–17.3°C) across all observation days and for both control and fouling pens. Thus, it did not differ between control and fouling pens, and it did not change prior to day0. However, pen temperature above the slatted floor increased with an increase in group size [*F*_(1, 234)_ = 13.03; *P* < 0.001].

## Discussion

The results indicate that pigs change their lying pattern prior to pen fouling with a gradual decline in lying on the solid floor followed by a delayed decline in temperature above the solid floor. However, these changes were only observed in pens without straw. Pigs in pens with straw seemed to prefer staying on the solid floor even close to an event of pen fouling.

### Changes in Pigs' Lying Pattern Prior to Fouling

Number of pigs lying in the pen did not change prior to fouling in the chosen observation periods, but number of pigs lying in the solid and slatted area did. As expected, fewer and fewer pigs rested on the solid floor, and more and more pigs rested on the slatted floor close to fouling; however, this only applied to pens without straw. Straw seemed to keep the pigs on the solid floor even when the floor became more and more soiled prior to the event. Both a solid floor area and straw may provide more comfort while resting compared to a slatted floor area. Thus, the majority of pigs rested in the solid floor area irrespective of straw being provided or not. However, straw may increase comfort while resting even further, delaying when the pigs will begin to move to the slatted area during the development of an event of fouling. Also, straw did not seem to remove the cause of fouling as almost half of the fouling events happened in pens provided with straw. Data from the present study were also used for a risk factor analysis that concluded that straw seemed to prevent fouling in the first half of the finisher period and to increase the risk of fouling in the second half of the finisher period (7). However, the current results suggest that getting the pigs to keep lying on the solid floor by means of comfortable bedding does not prevent fouling; although it may not develop into soiling of the entire solid floor and may give the farmer a longer time window for doing interventions in the pen to prevent further development of fouling in the pen.

Previous studies investigating fouling have tried to elucidate whether pigs first change their lying behaviour followed by a change in excretion behaviour or the other way around. Aarnink et al. (8) found a lower inflection temperature (start point) for excreting in the solid area than for lying in the slatted area, arguing that pigs change their excretion behaviour first. However, the inflection temperature for lying in the slatted area was measured at an area occupation of 100%. On the other hand, Huynh et al. (9) measured the inflection temperature for lying in the slatted area when the pigs started to change from lying in the solid area to the slatted area. They found a lower inflection temperature for lying in the slatted area than for excreting in the solid area. Results of the current study confirm both results. First, it showed that the pigs changed their lying behaviour prior to soiling half of the solid area. Second, it showed that only around 25% of the pigs were lying in the slatted area when half of the solid area was soiled, thus a 100% occupation of the slatted area was not seen prior to fouling. This argues that fouling develops gradually and, therefore, it may be possible to use these gradual changes for early detection and interventions to prevent serious pen fouling. The current study cannot further elucidate which behaviour the pigs change first but merely that a change in lying behaviour is seen prior to soiling half of the solid area.

The study confirms that pigs prefer to lie on the solid area compared to the slatted area (1), although this was not inferentially analysed. Pigs' lying location also seemed to depend on the group size and age with a lower percentage of pigs lying in the solid area with an increase in both parameters. These results make sense, as the larger the group size and age of the pigs, the more space the pigs occupy. Thus, the solid area may not offer enough room for all pigs that instead will lie on the drained and slatted areas. At a higher age, the pigs also produce more internal heat, thus lying more in lateral position or huddling less, resulting in each pig taking up even more space (10). This could also explain that an increase in age was followed by an increase in the percentage of pigs lying in the slatted area, as the slatted area was the cooler part of the pen. However, pigs lying posture was not recorded in the current study and thus, cannot further elucidate on this hypothesis.

### Changes in Pen Temperature Prior to Fouling

Pen temperatures also changed prior to an event of fouling but not as expected. Multiple studies have shown that high temperatures increase the degree of fouling [e.g., (10, 11)] and that a climate in the pen which is not optimal is the major cause of fouling (4). Thus, it was expected that pen temperatures, and especially when measured above the solid area, would increase prior to fouling. In contrast, the pen temperature measured above the solid area decreased. Further, this change seems parallel to pigs' change in lying behaviour prior to fouling. The causality can be discussed, but as the slatted area was colder it seems unlikely that a decrease in pen temperature above the solid floor would be followed by the pigs changing from lying in the solid area to lying in the slatted area. Thus, the results argue that the change in lying behaviour caused a change in pen temperature above the solid area as a result of fewer pigs heating up the solid area. The results also suggest that in the present study, fouling may be caused by other factors such as draught (12) or other subtle differences between the pens; although, no particular pattern was found across batches in the location of the fouling pens. These subtle differences between pens make it difficult for the farmer to control the climate, which is mainly done according to room temperature. The pen temperature seems to depend on the pigs' lying pattern. Thus, controlling the climate according to the pen temperature may not be optimal either. However, pen temperature may be able to signal or confirm changes in the behaviour of pigs, indicating problems with the climate of the single pen and thereby contributing to the overall climate control by for example controlling sprinklers at pen level.

### Early Detection of Fouling

To be an early detector of an event, the measure has to change prior to the event. Further this change cannot be seen in the pens not developing the event. According to this definition, both pigs' lying pattern and pen temperature measured above the solid area (pigs' resting area) have potential as early detectors of fouling, although only in pens without straw provided on the solid floor. More work is needed to develop an alarm system on-farm, including an automatic method to measure the lying pattern of pigs, e.g., by using image analysis, and the development of machine learning algorithms to predict fouling with both a high sensitivity and specificity. The cheapest and easiest implementation of such an alarm system would be to only include the pen temperature above the solid floor as an early detector. However, pen temperature can be affected by many other factors than merely pigs' lying pattern and thus, needs validation in a real-life setting including each day of the entire production period. Although, pen temperature has earlier been found to be a promising predictor of pen fouling (13). Also, to combine pigs' lying pattern and the pen temperature as early detectors could prove to perform even better than the separate single measures.

## Conclusions

Prior to an event of fouling, pigs changed their lying pattern with fewer and fewer pigs lying on the solid floor and more and more pigs lying on the slatted floor. Thus, pigs' lying pattern has potential as an early detector of pen fouling and as input for an early warning system, which can be measured using video surveillance and image analysis. Pen temperature measured above the solid floor also decreased prior to fouling. Thus, pen temperature also have potential as an early detector of pen fouling and perhaps with an even higher performance in combination with the changes seen in pigs' lying pattern prior to fouling.

## Ethics Statement

This study was carried out in accordance with the recommendations of Guidelines for Ethical Treatment of Animals in Applied Animal Behaviour and Welfare Research by the International Society of Applied Ethology (ISAE). The protocol was approved by the Danish Animal Experiments Inspectorate (Journal no. 2015-15-0201-00593).

## Author Contributions

All authors contributed to the design and conduction of the study. MB performed the literature study. MB and LP performed the preliminary descriptive and inferential analyses. ML performed the final statistical analysis and wrote the first draft of the manuscript. All authors contributed to manuscript revision and have read and approved the submitted version.

### Conflict of Interest Statement

The authors declare that the research was conducted in the absence of any commercial or financial relationships that could be construed as a potential conflict of interest.

## References

[1] Aarnink AJA vandenBerg AJ Keen A Hoeksma P Verstegen MWA Effect of slatted floor area on ammonia emission and on the excretory and lying behaviour of growing pigs. J Agric Eng Res. (1996) 64:299–310. 10.1006/jaer.1996.0071

[2] Hillmann E Mayer C Schrader L Lying behaviour and adrenocortical response as indicators of the thermal tolerance of pigs of different weights. Anim Welfare. (2004) 13:329–35.

[3] Smulders D Verbeke G Mormede P Geers R. Validation of a behavioral observation tool to assess pig welfare. Physiol Behav. (2006) 89:438–47. 10.1016/j.physbeh.2006.07.002 16904137

[4] Larsen MLV Bertelsen M Pedersen LJ. Review. Factors affecting fouling in conventional pens for slaughter pigs. Animal. (2018) 12:322–8. 10.1017/S.1751731117001586 28693639

[5] Larsen MLV Andersen HM-L Pedersen LJ. Which is the most preventive measure against tail damage in finisher pigs: tail docking, straw provision or lowered stocking density? Animal. (2018) 12:1260–7. 10.1017/S175173111700249X 29094665

[6] R Core Team (2017). R: A Language and Environment for Statistical Computing. Vienna: R Foundation for Statistical Computing. Available online at: https://www.R-project.org/

[7] Larsen MLV Bertelsen M Pedersen LJ How do stocking density and straw provision affect fouling in conventionally housed slaughter pigs? Livestock Sci. (2017) 205:1–4. 10.1016/j.livsci.2017.09.005

[8] Aarnink AJA Schrama JW Heetkamp MJW Stefanowska J Huynh TTT. Temperature and body weight affect fouling of pig pens. J Anim Sci. (2006) 84:2224–31. 10.2527/jas.2005-521 16864884

[9] Huynh TTT Aarnink AA Gerrits WJJ Heetkamp MJH Canh TT Spoolder HAM Thermal behaviour of growing pigs in response to high temperature and humidity. Appl Anim Behav Sci. (2005) 91:1–16. 10.1016/j.applanim.2004.10.020

[10] Spoolder HAM Aarnink AAJ Vermeer HM van Riel J Edwards SA Effect of increasing temperature on space requirements of group housed finishing pigs. Appl Anim Behav Sci. (2012) 138:229–39. 10.1016/j.applanim.2012.02.010

[11] Savary P Gygax L Wechsler B Hauser R Effect of a synthetic plate in the lying area on lying behaviour, degree of fouling and skin lesions at the leg joints of finishing pigs. Appl Anim Behav Sci. (2009) 118:20–7. 10.1016/j.applanim.2009.02.006

[12] Randall JM Armsby AW Sharp JR Cooling gradients across pens in a finishing piggery: *II.* Effects on excretory behavior. J Agric Eng Res. (1983) 28:247–59. 10.1016/0021-8634(83)90073-2

[13] Jensen DB Kristensen AR Temperature as a predictor of fouling and diarrhea in slaughter pigs. Livestock Sci. (2016) 183:1–3. 10.1016/j.livsci.2015.11.007

